# Supplementary material for: Over twenty years of publications in Ecology: Over-contribution of women reveals a new dimension of gender bias
Source: PLoS One. 2024 Sep 19;19(9):e0307813. doi: 10.1371/journal.pone.0307813 (PMC11412523; doi:10.1371/journal.pone.0307813)
Supplement: S1 File — Printed version of the R markdown code for the data analysis and complete results in html format. (HTML) [file pone.0307813.s001.html]

Codes for women over-contribution analyses


# Codes for women over-contribution analyses

#### CienciaFem

#### 22/9/2023

- 1. DATA PREPARATION
  - 1.1. Load the dataset EcologyAuthorshipsGender1999-2021
  - 1.2. Extract key information into independent objects
  - 1.3. Compilation of authors characterization table (DFautorxs)
  - 1.4. Frequency of authorship positions by gender
  - 1.5. Compilation of articles characterization table (DFpapers)
  - 1.6. Compilation of time series table (DFserieanual)
- 2. DATASET GRAPHIC OVERVIEW
- 3. OVERCONTRIBUTION ANALYSES
  - 3.1 Are women contributions higher than expected by chance?
  - 3.2. Comparing the centered WCI with the centered MCI
  - 3.3. Subset mix-gender articles only: Comparing the centered WCI with the centered MCI in mix-gender articles
- 4. Key numbers
- Packages references

In this document we share the R code to recreate the analyses and results of the article *Over-contribution of women in top-ranked journal reveals a new dimension of gender bias* by Fontanarrosa, Zarbá et al. 2024.

Packages

```
library(tidyverse)
library(magrittr)
library(knitr)
library(GGally)
library(cowplot)
library(ggthemes)
library(scales)
library(stats)
```

## 1. DATA PREPARATION

### 1.1. Load the dataset EcologyAuthorshipsGender1999-2021

```
#EcologyAuthorshipsGender1999-2021 dataset
info_papers <-  read.csv("EcologyAuthorshipsGender1999-2021.csv")
head(info_papers)
```

```
##    Año Vol_issue   aut_1   aut_2  aut_3  aut_4  aut_5  aut_6 aut_7 aut_8 aut_9
## 1 1999     80(1) M_10166 F_10244   <NA>   <NA>   <NA>   <NA>  <NA>  <NA>  <NA>
## 2 1999     80(1)  F_6241 F_10623   <NA>   <NA>   <NA>   <NA>  <NA>  <NA>  <NA>
## 3 1999     80(1)  M_3765   M_910 F_2822   <NA>   <NA>   <NA>  <NA>  <NA>  <NA>
## 4 1999     80(1)  M_2882  M_9734   M_65   <NA>   <NA>   <NA>  <NA>  <NA>  <NA>
## 5 1999     80(1)  F_2245  M_9581   <NA>   <NA>   <NA>   <NA>  <NA>  <NA>  <NA>
## 6 1999     80(1)  M_6049  M_6922 M_4883 M_4150 M_9178 M_5969  <NA>  <NA>  <NA>
##   aut_10 aut_11 aut_12 aut_13 aut_14 aut_15 aut_16 aut_17 aut_18 aut_19 aut_20
## 1   <NA>   <NA>   <NA>   <NA>   <NA>   <NA>   <NA>   <NA>   <NA>   <NA>   <NA>
## 2   <NA>   <NA>   <NA>   <NA>   <NA>   <NA>   <NA>   <NA>   <NA>   <NA>   <NA>
## 3   <NA>   <NA>   <NA>   <NA>   <NA>   <NA>   <NA>   <NA>   <NA>   <NA>   <NA>
## 4   <NA>   <NA>   <NA>   <NA>   <NA>   <NA>   <NA>   <NA>   <NA>   <NA>   <NA>
## 5   <NA>   <NA>   <NA>   <NA>   <NA>   <NA>   <NA>   <NA>   <NA>   <NA>   <NA>
## 6   <NA>   <NA>   <NA>   <NA>   <NA>   <NA>   <NA>   <NA>   <NA>   <NA>   <NA>
##   aut_21 aut_22 aut_23 aut_24 aut_25 aut_26 aut_27 aut_28 aut_29 aut_30 aut_31
## 1   <NA>   <NA>   <NA>   <NA>   <NA>   <NA>   <NA>   <NA>   <NA>   <NA>   <NA>
## 2   <NA>   <NA>   <NA>   <NA>   <NA>   <NA>   <NA>   <NA>   <NA>   <NA>   <NA>
## 3   <NA>   <NA>   <NA>   <NA>   <NA>   <NA>   <NA>   <NA>   <NA>   <NA>   <NA>
## 4   <NA>   <NA>   <NA>   <NA>   <NA>   <NA>   <NA>   <NA>   <NA>   <NA>   <NA>
## 5   <NA>   <NA>   <NA>   <NA>   <NA>   <NA>   <NA>   <NA>   <NA>   <NA>   <NA>
## 6   <NA>   <NA>   <NA>   <NA>   <NA>   <NA>   <NA>   <NA>   <NA>   <NA>   <NA>
##   aut_32 aut_33 aut_34 aut_35 aut_36 aut_37 aut_38 aut_39 aut_40 aut_41 aut_42
## 1   <NA>   <NA>   <NA>   <NA>   <NA>   <NA>   <NA>   <NA>   <NA>   <NA>   <NA>
## 2   <NA>   <NA>   <NA>   <NA>   <NA>   <NA>   <NA>   <NA>   <NA>   <NA>   <NA>
## 3   <NA>   <NA>   <NA>   <NA>   <NA>   <NA>   <NA>   <NA>   <NA>   <NA>   <NA>
## 4   <NA>   <NA>   <NA>   <NA>   <NA>   <NA>   <NA>   <NA>   <NA>   <NA>   <NA>
## 5   <NA>   <NA>   <NA>   <NA>   <NA>   <NA>   <NA>   <NA>   <NA>   <NA>   <NA>
## 6   <NA>   <NA>   <NA>   <NA>   <NA>   <NA>   <NA>   <NA>   <NA>   <NA>   <NA>
##   aut_43 aut_44 aut_45 aut_46 aut_47
## 1   <NA>   <NA>   <NA>   <NA>   <NA>
## 2   <NA>   <NA>   <NA>   <NA>   <NA>
## 3   <NA>   <NA>   <NA>   <NA>   <NA>
## 4   <NA>   <NA>   <NA>   <NA>   <NA>
## 5   <NA>   <NA>   <NA>   <NA>   <NA>
## 6   <NA>   <NA>   <NA>   <NA>   <NA>
```

### 1.2. Extract key information into independent objects

```
#authorship events
autorias <- na.omit(unlist(as.vector(info_papers[,3:49] )))

#authors
autorxs <-  unique(autorias)

#list of authors by article
listas_autorias <- apply(info_papers[,3:49], 1, function(x) {
  x[(nchar(x) > 0) & (!is.na(x))]}) 

#binary-coded list of authors by article: Women=1 & men=0 
listas_autorias1_0 <- lapply(listas_autorias, function(x) ifelse(str_detect(unlist(x), "F"), 1, 0 ))
```

Compute Women Contribution Index (WCI). First we compute authors’ contributions to each article based on their position in the authors list and the armonic weighting. Then, we compute the women contribution index per article as the sum of all contributions made by female authors only.

```
#contributions by author
contribuciones <- lapply(listas_autorias, function(x) prop.table(1/c(1:length(x))))

#Women Contribution Index
WCI<-c()
for(i in 1:length(listas_autorias1_0)){
  WCI<-c(WCI,sum(listas_autorias1_0[[i]]*contribuciones[[i]], na.rm = T))
}
```

Example:

```
#authors' list of article 4782
listas_autorias[[4728]]
```

```
##    aut_1    aut_2    aut_3    aut_4    aut_5    aut_6 
## "M_9907" "F_7053" "M_7458" "M_5687" "M_4874" "F_9621"
```

```
#authors' contributions of article 4782
contribuciones[[4728]]
```

```
## [1] 0.40816327 0.20408163 0.13605442 0.10204082 0.08163265 0.06802721
```

```
#Women Contribution Index (WCI) of article 4782
WCI[4728]
```

```
## [1] 0.2721088
```

### 1.3. Compilation of authors characterization table (DFautorxs)

For each author we measure: number of total authorship events, number of authorships as first, last, middle, and unique author, and their cumulative contributions.

```
#create empty objects to store temporal data
nombre <- c() #names of all authors per paper
primer <- c() #names of all first authors
ultimx <- c() #names of all last authors
solx <- c()   ##names of all solitary authors
cont <- c()   #contribution
pos_j <- c()  #position in authors' list
pos_clave <- c() #key position
indcont_autorx <- autorxs


#loop to extract position data
for (i in seq_along(listas_autorias)) { #iterate for each article
  
  if (length(listas_autorias[[i]])==1){  #is it a solitary-author article?
  solx <- c(solx, listas_autorias[[i]])  #extract name
  } else { #does the article have two or more authors?
  primer <- c(primer,listas_autorias[[i]][[1]]) #extract name 
  ultimx <- c(ultimx,listas_autorias[[i]][[length(listas_autorias[[i]])]]) #extract name
  }
  
  #register authors' position frequencies and contributions
  for (j in seq_along(listas_autorias[[i]])) { #iterate for each author in article i
    nombre <- c(nombre, listas_autorias[[i]][[j]]) 
    cont <- c(cont, contribuciones[[i]][[j]]) 
    pos_j <- c(pos_j,j)  
    
    if (length(listas_autorias[[i]])==1) { pos_clave <- c(pos_clave,"solx")
      } else if  ( length(listas_autorias[[i]])>1 & j==1) { pos_clave <- c(pos_clave,"1er")
      } else if  ( length(listas_autorias[[i]])>2 & j==2) { pos_clave <- c(pos_clave,"2do")
      } else if  ( length(listas_autorias[[i]])>3 & 2<j & j<length(listas_autorias[[i]])) { pos_clave <- c(pos_clave,"medio")
      } else if ( j==length(listas_autorias[[i]]) & length(listas_autorias[[i]])>1) { pos_clave <- c(pos_clave,"ult")}
  } 
  }
```

Compile authors information into a table

```
#count total number of authorships by each author
DFautorxs <- data.frame(AuthorCode = nombre,
                        republication = rep(1, length(nombre))) 
DFautorxs <- DFautorxs %$% aggregate(republication~ AuthorCode, FUN = sum) 
DFautorxs$Genero <- case_when(str_detect(DFautorxs$AuthorCode ,"F") ~ "Female",
                                         str_detect(DFautorxs$AuthorCode ,"M") ~ "Male")
DFautorxs <- DFautorxs [order(DFautorxs$republication,decreasing =TRUE),] #organize in decreasing order

#dissagregate number of authorships by position
DFautorxs <- DFautorxs %>% 
  left_join(as.data.frame(table(primer, dnn = list("AuthorCode")), responseName = "Primerx"))%>%
  left_join(as.data.frame(table(ultimx, dnn = list("AuthorCode")), responseName = "Ultimx"))%>%
  left_join(as.data.frame(table(solx, dnn = list("AuthorCode")), responseName = "Solx"))%>%
  mutate_each(funs(replace(., which(is.na(.)), 0))) %>%
  mutate (Medio=republication-(Primerx+Ultimx+Solx))
```

Cumulative contribution by authorship position for each author

```
df <- data.frame(AuthorCode=nombre,PosFreq=pos_j, contrib_tot=cont, pos_clave) #create temporal data frame

#total cumulative contributions
DFautorxs <- DFautorxs%>% left_join( df %$% aggregate(contrib_tot~ AuthorCode, FUN = sum)) 

#cumulative contributions as solitary author
DFautorxs <- DFautorxs%>% left_join(filter(df, pos_clave== "solx") %$% 
  aggregate(contrib_tot~ AuthorCode, FUN = sum)%>%
  rename("contrib_solx"="contrib_tot"))

#cumulative contributions as first author
DFautorxs <- DFautorxs%>% left_join(filter(df, pos_clave== "1er") %$% 
  aggregate(contrib_tot~ AuthorCode, FUN = sum)%>%
  rename("contrib_1er"="contrib_tot"))

#cumulative contributions as second author
DFautorxs <- DFautorxs%>% left_join(filter(df, pos_clave== "2do") %$% 
  aggregate(contrib_tot~ AuthorCode, FUN = sum)%>%
  rename("contrib_2do"="contrib_tot"))

#cumulative contributions as middle author
DFautorxs <- DFautorxs%>% left_join(filter(df, pos_clave== "medio") %$% 
  aggregate(contrib_tot~ AuthorCode, FUN = sum)%>%
  rename("contrib_medio"="contrib_tot"))

#cumulative contributions as last author
DFautorxs <- DFautorxs%>% left_join(filter(df, pos_clave== "ult") %$% 
  aggregate(contrib_tot~ AuthorCode, FUN = sum)%>%
  rename("contrib_ult"="contrib_tot"))


#inspect the table
head(DFautorxs)
```

```
##   AuthorCode republication Genero Primerx Ultimx Solx Medio contrib_tot
## 1    M_10765            27   Male       7      8    0    12    6.673785
## 2     M_7576            27   Male       6     15    0     6    7.580503
## 3      M_829            27   Male       4     15    0     8    7.032977
## 4     M_9499            26   Male       7     16    0     3    7.012158
## 5     M_4683            25   Male       2      7    0    16    5.401367
## 6      M_853            24   Male       3     11    7     3   12.661352
##   contrib_solx contrib_1er contrib_2do contrib_medio contrib_ult
## 1           NA   2.8131855   1.6499103     0.6387676    1.571922
## 2           NA   3.0480336   0.6957870     0.4800000    3.356682
## 3           NA   2.3030303   0.9927273     0.6400000    3.097220
## 4           NA   3.1349788   0.5454545     0.1600000    3.171725
## 5           NA   0.9179562   2.3860983     0.6157823    1.481531
## 6            7   1.6202845   0.5127273     0.1074156    3.420925
```

### 1.4. Frequency of authorship positions by gender

Compute the frequencies

```
#First, transform the 'listas_autorias' list into a matrix. Columns are authorships' positions
n.obs <- sapply(listas_autorias1_0, length)
seq.max <- seq_len(max(n.obs))
mat <- t(sapply(listas_autorias1_0, "[", i = seq.max))

#Then, calculate the number of female and male authors by authorship position
countbycol <- NULL
for(u in 1:ncol(mat)){  #for each authorship position
  x <- table(mat[ ,u])
  countbycol <- c(countbycol, x[1], x[2]) #saves the number of female and male authors by category
}


#generate a matrix
posicion_autoria <- data.frame(posautor = rep(1:ncol(mat), each = 2),
                      sex = rep(c('male','female'), ncol(mat)), # = order 0,1
                      naut = countbycol)
posicion_autoria$naut[is.na(posicion_autoria$naut)] <- 0

#total authorships by gender
totMALE <- posicion_autoria %>% filter(sex=="male")%>%select(naut)%>%sum(na.rm = T)
totFEMALE <- posicion_autoria %>% filter(sex=="female")%>%select(naut)%>%sum(na.rm = T)
```

Arrange in a table of authorship position relative frequencies by gender

```
#Authorship frequencies relative to the total for each gender
posicion_autoria <- posicion_autoria%>% mutate( #frequency relative to the total for the gender
  FreqRelativa=case_when(
  sex=="male"~naut/totMALE,
  sex=="female"~naut/totFEMALE

))

head(posicion_autoria)
```

```
##   posautor    sex naut FreqRelativa
## 1        1   male 3507    0.2662870
## 2        1 female 1625    0.3207026
## 3        2   male 3448    0.2618071
## 4        2 female 1168    0.2305112
## 5        3   male 2318    0.1760061
## 6        3 female  846    0.1669627
```

### 1.5. Compilation of articles characterization table (DFpapers)

Gender of authors by position

```
#total authorships
total_aut <- sapply(listas_autorias1_0, FUN = function(x) length(x))

#total female authors
total_mujeres <- c()
for (i in seq_along(listas_autorias1_0))  {total_mujeres <- c(total_mujeres, sum(listas_autorias1_0[[i]], na.rm = T))}

#total male authors
total_hombres <- c()
p_h <- lapply(listas_autorias1_0, FUN = 
                function(x) {
                  ifelse(x == 1, 0,
                         ifelse(x == 0, 1, NA))})
for (i in seq_along(p_h)) {total_hombres <- c(total_hombres, sum(p_h[[i]], na.rm = T))}


#female proportion
proporcion <- c()
for (i in seq_along(listas_autorias1_0)) { proporcion <- c(proporcion, mean(listas_autorias1_0[[i]], na.rm = T))}

#first author gender
pos_1er <- c()
for (i in seq_along(listas_autorias1_0)) { 
if (listas_autorias1_0[[i]][1] == 1) { 
    pos_1er <- c(pos_1er, "mujer")  
  } else {                          
    pos_1er <- c(pos_1er, "varon")  
  }
}

#gender of last author (for articles with two or more authors)
pos_ult <- c()
for (i in seq_along(listas_autorias1_0)) {
  naut=length(listas_autorias1_0[[i]])     
  if (naut == 1) { 
    pos_ult <- c(pos_ult, NA)  
  } else if (listas_autorias1_0[[i]][naut] == 1) { 
    pos_ult <- c(pos_ult, "mujer") 
  } else {                         
    pos_ult <- c(pos_ult, "varon") 
  }
}


#gender of second author (for articles with three or more authors)
pos_2do <- c()
for (i in seq_along(listas_autorias1_0)) {
  if (length(listas_autorias1_0[[i]]) < 3 ) { 
    pos_2do <- c(pos_2do, NA)         
  } else if (listas_autorias1_0[[i]][2] == 1) {
    pos_2do <- c(pos_2do, "mujer")
  } else {                         
    pos_2do <- c(pos_2do, "varon") 
  }
}
```

Compile the data frame

```
DFpapers <- data.frame(year = info_papers$Año,
                 aut_tot = total_aut,
                 mujeres_tot = total_mujeres,
                 varones_tot = total_hombres,
                 mujeres_prop = proporcion,
                 varones_prop = 1 - proporcion,
                 sex_1er = pos_1er,
                 sex_2do = pos_2do,
                 sex_ult = pos_ult,
                 WCI = WCI)
```

Add gender homogeneity data: differentiate articles where all authors share the same gender from mixed-gender articles

```
DFpapers <- DFpapers %>%
  mutate( monogenero=case_when( 
    aut_tot==mujeres_tot ~ "mono_F",
    aut_tot==varones_tot ~ "mono_M",
    aut_tot!=varones_tot & aut_tot!=mujeres_tot ~ "mixto"))%>%
  mutate_at("monogenero", as.factor)


head(DFpapers)
```

```
##   year aut_tot mujeres_tot varones_tot mujeres_prop varones_prop sex_1er
## 1 1999       2           1           1    0.5000000    0.5000000   varon
## 2 1999       2           2           0    1.0000000    0.0000000   mujer
## 3 1999       3           1           2    0.3333333    0.6666667   varon
## 4 1999       3           0           3    0.0000000    1.0000000   varon
## 5 1999       2           1           1    0.5000000    0.5000000   mujer
## 6 1999       6           0           6    0.0000000    1.0000000   varon
##   sex_2do sex_ult       WCI monogenero
## 1    <NA>   mujer 0.3333333      mixto
## 2    <NA>   mujer 1.0000000     mono_F
## 3   varon   mujer 0.1818182      mixto
## 4   varon   varon 0.0000000     mono_M
## 5    <NA>   varon 0.6666667      mixto
## 6   varon   varon 0.0000000     mono_M
```

### 1.6. Compilation of time series table (DFserieanual)

```
DFserieanual <- DFpapers %>% 
  group_by(year) %>%  #for each year we calculate:
  summarise(
    n_papers=n(), #number of articles
    aut_tot_sum=sum(aut_tot), #number of authorships
    
    #articles averages (and standard deviations):
    aut_tot_prom=mean(aut_tot), #mean authors by article
    mujeres_prop_prom=mean(mujeres_prop), #mean proportion of female authors by article
    WCI_prom=mean(WCI), #mean Women Contribution Index by article
    aut_tot_sd=sd(aut_tot),
    mujeres_prop_sd=sd(mujeres_prop),
    WCI_sd=sd(WCI),
    
    #position proportions by gender
    F_1er_prop=sum(sex_1er=="mujer")/ n_papers, #proportion of articles with female first author
    F_ult_prop=sum(sex_ult=="mujer", na.rm = T)/ n_papers, #proportion of articles with female last author
    F_sola_prop=sum(sex_1er=="mujer" & aut_tot==1)/ n_papers, #proportion of articles with female solitary author
    M_solo_prop=sum(sex_1er=="varon" & aut_tot==1)/ n_papers, #proportion of articles with male solitary author
    mono_F_prop=sum(monogenero=="mono_F")/ n_papers, #proportion of articles with female authors only
    mono_M_prop=sum(monogenero=="mono_M")/ n_papers, #proportion of articles with male authors only
    mixto_prop=sum(monogenero=="mixto")/ n_papers, #proportion of articles with mixed-gender authors
  )

head(DFserieanual)
```

```
## # A tibble: 6 × 16
##    year n_papers aut_tot_sum aut_tot_prom mujeres_prop_prom WCI_prom aut_tot_sd
##   <int>    <int>       <int>        <dbl>             <dbl>    <dbl>      <dbl>
## 1  1999      205         524         2.56             0.181    0.185       1.32
## 2  2000      250         649         2.60             0.222    0.222       1.34
## 3  2001      232         602         2.59             0.223    0.238       1.40
## 4  2002      264         681         2.58             0.207    0.210       1.41
## 5  2003      253         718         2.84             0.187    0.198       1.89
## 6  2004      147         334         2.27             0.226    0.226       1.25
## # ℹ 9 more variables: mujeres_prop_sd <dbl>, WCI_sd <dbl>, F_1er_prop <dbl>,
## #   F_ult_prop <dbl>, F_sola_prop <dbl>, M_solo_prop <dbl>, mono_F_prop <dbl>,
## #   mono_M_prop <dbl>, mixto_prop <dbl>
```

## 2. DATASET GRAPHIC OVERVIEW

Total authorships by year

```
gg <- DFserieanual%>%
  ggplot(aes(year, aut_tot_sum)) +
  geom_bar( stat = "identity")+
  labs (y="Total authorships", x="Year")+
  themeCieciaFem()
gg
```

Number of authors per article

```
gg <- DFpapers %>%
  ggplot(aes(aut_tot))+
  geom_histogram(binwidth = 1, fill="grey70", col="black")+
  labs(x="Authorships per author", y="Frequency")+
  themeCieciaFem()
gg
```

```
table(DFpapers$aut_tot)
```

```
## 
##    1    2    3    4    5    6    7    8    9   10   11   12   13   14   15   16 
##  516 1452 1189  808  505  245  140   91   60   36   17   21   10    9    4    1 
##   17   18   20   21   22   23   25   26   27   29   33   35   47 
##    3    5    3    4    3    2    2    1    1    1    1    1    1
```

Contingency table of most frequent articles’ authorship structure

```
DFpapers %>% group_by(aut_tot,monogenero)%>%
  summarise(freq=n())%>%
  arrange(-freq) %>%
  head(10)
```

```
## # A tibble: 10 × 3
## # Groups:   aut_tot [6]
##    aut_tot monogenero  freq
##      <int> <fct>      <int>
##  1       2 mono_M       842
##  2       3 mixto        686
##  3       4 mixto        531
##  4       2 mixto        480
##  5       3 mono_M       466
##  6       1 mono_M       407
##  7       5 mixto        391
##  8       4 mono_M       267
##  9       6 mixto        215
## 10       2 mono_F       130
```

Average authors by article by year

```
gg <- DFpapers %>% ggplot(aes(year, aut_tot)) +
  stat_summary(fun = "mean", geom = "point", position = "dodge") +
  stat_summary(fun.data = mean_cl_boot, geom = "errorbar", position = position_dodge()) +
  theme(axis.text.x = element_text(angle = 45, vjust = 1, hjust = 1)) +
  labs(y="Average authors per article", x="Year")+
  themeCieciaFem()
gg
```

Proportion of female authorships by year

```
gg <- DFpapers %>% ggplot(aes(year, mujeres_prop)) +
  stat_summary(fun = "mean", geom = "point", position = "dodge") +
  stat_summary(fun.data = mean_cl_boot, geom = "errorbar", position = position_dodge()) +
  theme(axis.text.x = element_text(angle = 45, vjust = 1, hjust = 1)) +
  labs(y="Proportion of female authorships", x="Year")+
  themeCieciaFem()
gg
```

Mean WCI by year

```
gg <- DFpapers %>% ggplot(aes(year, WCI)) +
  stat_summary(fun = "mean", geom = "point", position = "dodge") +
  stat_summary(fun.data = mean_cl_boot, geom = "errorbar", position = position_dodge()) +
  theme(axis.text.x = element_text(angle = 45, vjust = 1, hjust = 1)) +
  labs(y="WCI", x="Year")+
  themeCieciaFem()
gg
```

Gender proportion pie chart

```
DFcirculos <- data.frame(Genero=c("Woman","Men"),
                                 UniqueAutorxs=c(prop.table(table(DFautorxs$Genero))[[1]],prop.table(table(DFautorxs$Genero))[[2]]),
                                 TotalAutorias=c(totFEMALE/(totFEMALE+totMALE),totMALE/(totFEMALE+totMALE)))%>%
          pivot_longer(!Genero, names_to = "Autorxs", values_to = "Conteo")%>%
          rbind(data.frame(Genero= c("Woman", "Men"), Autorxs=c("TopRanked50", "TopRanked50"), Conteo=as.numeric(prop.table(table(DFautorxs[1:50,3])))))
        
        gg <- DFcirculos[c(5,6,2,4,1,3),]%>%
          ggplot(aes(x = Autorxs, y = Conteo, fill = Genero)) +
          geom_col() +
          scale_fill_manual(values = c("#fbb726ff","#542487ff") ) +
          coord_polar("y")+
          theme_minimal()
gg
```

Gender pie chart in numbers

```
DFcirculos[c(5,6,2,4,1,3),]
```

```
## # A tibble: 6 × 3
##   Genero Autorxs       Conteo
##   <chr>  <chr>          <dbl>
## 1 Woman  TopRanked50    0.14 
## 2 Men    TopRanked50    0.86 
## 3 Woman  TotalAutorias  0.278
## 4 Men    TotalAutorias  0.722
## 5 Woman  UniqueAutorxs  0.319
## 6 Men    UniqueAutorxs  0.681
```

Ranking top 100

```
gg <- DFautorxs[1:100,]%>%
          ggplot(aes(x=reorder(AuthorCode, republication),republication, fill=Genero))+
          geom_bar(stat = "identity")+
          scale_fill_manual(values = c("#542487ff","#fbb726ff") ) +
          labs(title="Ranking top 100 authors with more authorship events", x="Authors", y="Number of authorship events")+
          coord_flip()+
  themeCieciaFem()
gg
```

Re-publication histogram

```
gg <- DFautorxs %>%
  filter(republication>1)%>%
  ggplot(aes(republication))+
  geom_histogram()+
  labs(x="Authorships per author", y="Frequency")+
  themeCieciaFem()
gg
```

Frequency of authorship position by gender

```
gg <- ggplot(posicion_autoria, aes(fill = sex, y = naut, x = posautor)) +
  geom_bar(position = "dodge", stat = "identity") +
  labs(x="Authorship position", y="Total authors")+
  scale_fill_manual(values = c("#542487ff","#fbb726ff") )+
  themeCieciaFem()
gg
```

Time series of proportion of mix-gender, exclusively male and exclusively female authored articles

```
gg <- DFserieanual %>%
  pivot_longer(cols=mono_F_prop:mixto_prop, names_to="Type", values_to="Freq")%>%
  
  ggplot(aes(year, Freq*n_papers, fill=Type)) +
  geom_bar( stat = "identity", position = "stack")+
  labs (y="Number of articles", x="Year")+
  scale_fill_manual(values = c("#51a7ed","#542487ff", "#fbb726ff") ) +
  themeCieciaFem()
gg
```

Histogram of number of authors in mono-gender articles

```
gg <- DFpapers %>%
  filter(monogenero!="mixto")%>%
  ggplot(aes(aut_tot))+
  geom_histogram(col="white", binwidth=1 )+
  labs(x="Number of authors per article", y="Frequency")+
  facet_wrap("monogenero", scales = "free_y")+
  themeCieciaFem()
gg
```

```
#Female mono-gender articles - number of authors per article
round(prop.table(table(DFpapers %>%
  filter(monogenero=="mono_F")%>%
  select(aut_tot)))*100,2)
```

```
## aut_tot
##     1     2     3     4     5 
## 37.46 44.67 12.71  3.44  1.72
```

```
#Male mono-gender articles - number of authors per article
round(prop.table(table(DFpapers %>%
  filter(monogenero=="mono_M")%>%
  select(aut_tot)))*100,2)
```

```
## aut_tot
##     1     2     3     4     5     6     7     8     9    10    11    12    13 
## 18.86 39.02 21.59 12.37  5.05  1.39  0.74  0.23  0.37  0.19  0.05  0.05  0.09
```

## 3. OVERCONTRIBUTION ANALYSES

### 3.1 Are women contributions higher than expected by chance?

We compared the WCI calculated for our dataset (WCI\_observado) against the WCI we would expect to find in a scenario with no gender bias in the authorship positions, given the existing gender ratios per article (WCI\_proporcion\_F, null scenario). The WCI\_proporcion\_F was computed as the proportion of women in the author list, which is, in turn, equivalent to run infinite simulated scenarios where we exchange the position of female and male authors within each article’s authors list, maintaining the gender ratio (WCI\_simulados).

#### Expected WCI

We computed the expected WCI (WCI\_proporcion\_F) as the proportion of women in the author lists

```
WCI_proporcion_F <- unlist(lapply(listas_autorias1_0, mean))
```

#### Simulated WCI

We ran 10000 simulations of scenarios with no gender bias in the authorship positions and recorded the total WCI per simulated scenario, calculated as the sum of the WCI of all articles

```
#simulation of unbiased scenarios
set.seed(862)
WCI_simulados<-replicate(10000, sum(unlist(lapply(listas_autorias1_0, sample))*unlist(contribuciones), na.rm = T))
```

#### Hypothesis test against unbiased scenarios

We sum the WCI across all articles (total\_WCI) and compare the expected and observed WCI values

Sum of WCI across all 5132 articles

```
total_WCI_observado <- sum(WCI)
total_WCI_proporcion_F <- sum(WCI_proporcion_F)
total_WCI_simulado <- mean(WCI_simulados)

c(total_WCI_observado,total_WCI_proporcion_F, total_WCI_simulado)
```

```
## [1] 1456.080 1360.490 1360.626
```

How likely is it that this difference occurs?

```
# this proportion represents the p value
mean(WCI_simulados>total_WCI_observado)
```

```
## [1] 0
```

```
# how many times was the simulated total WCI higher than the observed WCI across the 10000 iterations?
table(WCI_simulados>total_WCI_observado)
```

```
## 
## FALSE 
## 10000
```

```
# in how many of the articles the observed WCI was higher than the expected WCI based on gender ratio?
prop.table(table(WCI > WCI_proporcion_F))
```

```
## 
##    FALSE     TRUE 
## 0.722915 0.277085
```

Looking at the article level, the observed WCI was higher than the WCI derived from gender ratios in 72.29% of the articles in our data set. However, at the data set level, we found that not in none of the 10,000 simulated scenarios was the total WCI higher than the total WCI observed in our data.

Density plot

```
gg <- ggplot(as.data.frame(WCI_simulados)) +
  geom_density(aes(x = WCI_simulados), color = "grey60", fill = "grey80") +
  geom_vline(xintercept = mean(WCI_simulados), color = "grey20", size=1)+
  geom_vline(xintercept = (mean(WCI_simulados)+sd(WCI_simulados)), color = "grey20", linetype="dashed")+
  geom_vline(xintercept = (mean(WCI_simulados)-sd(WCI_simulados)), color = "grey20", linetype="dashed")+
  geom_vline(xintercept = sum(WCI), color = "mediumpurple3", size=2)+
  labs(x="Women contribution index (WCI) in null scenarios")+
  scale_color_manual(values = c("#542487ff","#fbb726ff") ) +
  themeCieciaFem()
gg
```

### 3.2. Comparing the centered WCI with the centered MCI

```
#MCI
MCI=1-WCI
MCI_proporcion_M <- 1-WCI_proporcion_F

#centered contributions
WCI_centered <- WCI - WCI_proporcion_F
MCI_centered <- MCI - MCI_proporcion_M
```

Test Kolmogorov–Smirnov

```
test <- ks.test(WCI_centered,MCI_centered)
test
```

```
## 
##  Asymptotic two-sample Kolmogorov-Smirnov test
## 
## data:  WCI_centered and MCI_centered
## D = 0.13971, p-value < 2.2e-16
## alternative hypothesis: two-sided
```

Quantile-quantile plot comparing the centered MCI (X-axis) vs the centered WCI (Y-axis)

```
set.seed(54) 
subset1_M <- sample(1:nrow(DFpapers), round(nrow(DFpapers)/2,0) ) #subset for MCI calculations
subset2_F <- setdiff(1:nrow(DFpapers), subset1_M) #subset for WCI calculations
 
#set the color ramp
jet.colors <- colorRamp(c("blue", "cyan", "yellow", "red"))

#quantile-quantile plot
qqplot(MCI_centered[subset1_M], WCI_centered[subset2_F],  type = "l", xlab = "Centered MCI", ylab = "Centered WCI") ;  
abline(a = 0, b = 1, lty = "dotted", lwd = 1.5)
x <- quantile(MCI_centered[subset1_M], p = seq(0,1, 0.01))
y <- quantile(WCI_centered[subset2_F], p = seq(0,1, 0.01))
points(x, y, bg =  rgb(jet.colors(seq(0, 1, 0.01))/255), pch = 21, col = "gray", cex = 1)
text(0, 0.4, "order-p quantile", font = 4, adj = c(0.5, 0.5))
plot3D::colkey (col = rgb(jet.colors(seq(0, 1, 0.01))/255), side = 3, add = TRUE, clim = c(0, 1),  
          at = seq(0, 1, 0.2), labels = as.character(round(seq(0, 1, 0.2), 1)))
```

### 3.3. Subset mix-gender articles only: Comparing the centered WCI with the centered MCI in mix-gender articles

Filter and split the data set

```
dat <- filter(DFpapers, monogenero=="mixto") # N=2683

# observed contribution index
WCI_mixto <- c(dat$WCI)
MCI_mixto=1-WCI_mixto

# contribution index based on gender proportions per article
WCI_proporcion_F_mixto <- c(dat$mujeres_prop)
MCI_proporcion_M_mixto <- 1-WCI_proporcion_F_mixto

#centered contribution index
WCI_centered <- WCI_mixto - WCI_proporcion_F_mixto
MCI_centered <- MCI_mixto - MCI_proporcion_M_mixto

#split data in two
set.seed(54) 
subset1_M <- sample(1:nrow(dat), round(nrow(dat)/2,0) ) #subset for MCI calculations
subset2_F <- setdiff(1:nrow(dat), subset1_M) #subset for WCI calculations
```

Quantile-quantile plot comparing the MCI\_centered and the WCI\_centered

```
qqplot(MCI_centered[subset1_M], WCI_centered[subset2_F],  type = "l", xlab = "Centered MCI", ylab = "Centered WCI") ;  
abline(a = 0, b = 1, lty = "dotted", lwd = 1.5)
x <- quantile(MCI_centered[subset1_M], p = seq(0,1, 0.01))
y <- quantile(WCI_centered[subset2_F], p = seq(0,1, 0.01))
points(x, y, bg =  rgb(jet.colors(seq(0, 1, 0.01))/255), pch = 21, col = "gray", cex = 1)
text(0, 0.4, "order-p quantile", font = 4, adj = c(0.5, 0.5))
plot3D::colkey (col = rgb(jet.colors(seq(0, 1, 0.01))/255), side = 3, add = TRUE, clim = c(0, 1),  
          at = seq(0, 1, 0.2), labels = as.character(round(seq(0, 1, 0.2), 1)))
```

## 4. Key numbers

- Number of articles = 5132
- Number of authors = 11236
- Number of authorships = 18237
- Number of female authors = 3589 (31.94 %)
- Number of female authorships = 5074 (27.82 %)
- Average number of authors per paper = 3.5535853
- Average republication value = 1.6230865
- Average republication value of women = 1.4137643
- Average republication value of men = 1.7213286
- Women proportion among the 100 top-publishing authors =0.15
- Women proportion among the 50 top-publishing authors = 0.14
- Women proportion among the 25 top-publishing authors = 0.04
- Observed total WCI = 1456.0797045
- Expected total WCI = 1360.4903529
- Simulated total WCI = 1360.6264333
- Standard deviation of simulated WCI = 7.7473764
- Max simulated WCI = 1388.2291734

Subset mix-gender articles only

## Packages references

```
citation()
```

```
## To cite R in publications use:
## 
##   R Core Team (2023). _R: A Language and Environment for Statistical
##   Computing_. R Foundation for Statistical Computing, Vienna, Austria.
##   <https://www.R-project.org/>.
## 
## A BibTeX entry for LaTeX users is
## 
##   @Manual{,
##     title = {R: A Language and Environment for Statistical Computing},
##     author = {{R Core Team}},
##     organization = {R Foundation for Statistical Computing},
##     address = {Vienna, Austria},
##     year = {2023},
##     url = {https://www.R-project.org/},
##   }
## 
## We have invested a lot of time and effort in creating R, please cite it
## when using it for data analysis. See also 'citation("pkgname")' for
## citing R packages.
```

```
citation("tidyverse")
```

```
## To cite package 'tidyverse' in publications use:
## 
##   Wickham H, Averick M, Bryan J, Chang W, McGowan LD, François R,
##   Grolemund G, Hayes A, Henry L, Hester J, Kuhn M, Pedersen TL, Miller
##   E, Bache SM, Müller K, Ooms J, Robinson D, Seidel DP, Spinu V,
##   Takahashi K, Vaughan D, Wilke C, Woo K, Yutani H (2019). "Welcome to
##   the tidyverse." _Journal of Open Source Software_, *4*(43), 1686.
##   doi:10.21105/joss.01686 <https://doi.org/10.21105/joss.01686>.
## 
## A BibTeX entry for LaTeX users is
## 
##   @Article{,
##     title = {Welcome to the {tidyverse}},
##     author = {Hadley Wickham and Mara Averick and Jennifer Bryan and Winston Chang and Lucy D'Agostino McGowan and Romain François and Garrett Grolemund and Alex Hayes and Lionel Henry and Jim Hester and Max Kuhn and Thomas Lin Pedersen and Evan Miller and Stephan Milton Bache and Kirill Müller and Jeroen Ooms and David Robinson and Dana Paige Seidel and Vitalie Spinu and Kohske Takahashi and Davis Vaughan and Claus Wilke and Kara Woo and Hiroaki Yutani},
##     year = {2019},
##     journal = {Journal of Open Source Software},
##     volume = {4},
##     number = {43},
##     pages = {1686},
##     doi = {10.21105/joss.01686},
##   }
```

```
citation("magrittr")
```

```
## To cite package 'magrittr' in publications use:
## 
##   Bache S, Wickham H (2022). _magrittr: A Forward-Pipe Operator for R_.
##   R package version 2.0.3,
##   <https://CRAN.R-project.org/package=magrittr>.
## 
## A BibTeX entry for LaTeX users is
## 
##   @Manual{,
##     title = {magrittr: A Forward-Pipe Operator for R},
##     author = {Stefan Milton Bache and Hadley Wickham},
##     year = {2022},
##     note = {R package version 2.0.3},
##     url = {https://CRAN.R-project.org/package=magrittr},
##   }
```

```
citation("knitr")
```

```
## To cite package 'knitr' in publications use:
## 
##   Xie Y (2023). _knitr: A General-Purpose Package for Dynamic Report
##   Generation in R_. R package version 1.45, <https://yihui.org/knitr/>.
## 
##   Yihui Xie (2015) Dynamic Documents with R and knitr. 2nd edition.
##   Chapman and Hall/CRC. ISBN 978-1498716963
## 
##   Yihui Xie (2014) knitr: A Comprehensive Tool for Reproducible
##   Research in R. In Victoria Stodden, Friedrich Leisch and Roger D.
##   Peng, editors, Implementing Reproducible Computational Research.
##   Chapman and Hall/CRC. ISBN 978-1466561595
## 
## To see these entries in BibTeX format, use 'print(<citation>,
## bibtex=TRUE)', 'toBibtex(.)', or set
## 'options(citation.bibtex.max=999)'.
```

```
citation("GGally")
```

```
## To cite package 'GGally' in publications use:
## 
##   Schloerke B, Cook D, Larmarange J, Briatte F, Marbach M, Thoen E,
##   Elberg A, Crowley J (2024). _GGally: Extension to 'ggplot2'_. R
##   package version 2.2.1, <https://CRAN.R-project.org/package=GGally>.
## 
## A BibTeX entry for LaTeX users is
## 
##   @Manual{,
##     title = {GGally: Extension to 'ggplot2'},
##     author = {Barret Schloerke and Di Cook and Joseph Larmarange and Francois Briatte and Moritz Marbach and Edwin Thoen and Amos Elberg and Jason Crowley},
##     year = {2024},
##     note = {R package version 2.2.1},
##     url = {https://CRAN.R-project.org/package=GGally},
##   }
```

```
citation("cowplot")
```

```
## To cite package 'cowplot' in publications use:
## 
##   Wilke C (2024). _cowplot: Streamlined Plot Theme and Plot Annotations
##   for 'ggplot2'_. R package version 1.1.3,
##   <https://CRAN.R-project.org/package=cowplot>.
## 
## A BibTeX entry for LaTeX users is
## 
##   @Manual{,
##     title = {cowplot: Streamlined Plot Theme and Plot Annotations for 'ggplot2'},
##     author = {Claus O. Wilke},
##     year = {2024},
##     note = {R package version 1.1.3},
##     url = {https://CRAN.R-project.org/package=cowplot},
##   }
```
