# Supplementary material for: Over twenty years of publications in Ecology: Over-contribution of women reveals a new dimension of gender bias
Source: PLoS One. 2024 Sep 19;19(9):e0307813. doi: 10.1371/journal.pone.0307813 (PMC11412523; doi:10.1371/journal.pone.0307813)
Supplement: S2 File — In this appendix, we present the rationale behind our methodological decision to consider the last author as the one who contributes the least. We believe this decision minimizes the likelihood of error in quantifying the last author contribution. Additionally, we propose potential methodological alternatives that the Women Contribution Index allows for when deemed appropriate by the researcher. (PDF) [file pone.0307813.s002.pdf]

Supporting Information for:

Over twenty years of publications in Ecology: Over-contribution of Women reveals a new dimension of gender bias

Gabriela Fontanarrosa[1¶], Lucía Zarbá[2¶], Valeria Aschero[3], Daniel Andrés Dos Santos[1, 4], M. Gabriela Nuñez Montellano[5], Maia C. Plaza Behr[5], Natalia Schroeder[6,7], Silvia Beatriz Lomáscolo[5], María Elisa Fanjul[4,8], A. Carolina Monmany Garzia[5], Marisa, Alvarez[9,10], Agustina Novillo[1], María José Lorenzo Pisarello[11], Romina Elisa D'Almeida[12], Mariana Valoy[8], Andrés Felipe Ramírez-Mejía[5], Daniela Rodríguez[6,7], Celina Reynaga[1], María Leonor Sandoval Salinas[13,14], Verónica Chillo[15] & María Piquer-Rodríguez[16] *

[1] Instituto de Biodiversidad Neotropical (IBN), Consejo Nacional de Investigaciones Científicas y Técnicas (CONICET), Facultad de Ciencias Naturales e Instituto Miguel Lillo, Universidad Nacional de Tucumán (UNT), Yerba Buena, Tucumán, Argentina
[2] Instituto de Investigaciones Territoriales y Tecnológicas para la Producción del Hábitat UNT-CONICET, Tucumán, Argentina.
[3] Instituto Argentino de Nivología, Glaciología y Ciencias Ambientales (IANIGLA), CONICET, Universidad Nacional de Cuyo (UNCuyo), Argentina
[4] Instituto Vertebrados, Zoología, Fundación Miguel Lillo. Facultad de Ciencias Naturales e Instituto Miguel Lillo. Universidad Nacional de Tucumán, Argentina.
[5] Instituto de Ecología Regional (IER), Universidad Nacional de Tucumán (UNT)- Consejo Nacional de Investigaciones Científicas y Técnicas (CONICET), Tucumán, Argentina
[6] Instituto Argentino de Investigaciones de las Zonas Áridas (IADIZA), CCT-CONICET
[7] Facultad de Ciencias Agrarias, Universidad Nacional de Cuyo, Mendoza, Argentina
[8] Fundación Miguel Lillo, Tucumán, Argentina
[9] Universidad Nacional de Tucumán, Argentina (UNT)
[10] Universidad Nacional de Santiago del Estero, Argentina (UNSE)
[11] Centro de Referencia para Lactobacilos CCT NoA Sur. Consejo Nacional de Investigaciones Científicas y Técnicas (CONICET)h
[12] Instituto Superior de Investigaciones Biológicas (INSIBIO). CCT NoA Sur. Consejo Nacional de Investigaciones Científicas y Técnicas (CONICET)
[13] Instituto de Investigación en Luz, Ambiente y Visión (ILAV), CONICET-UNT
[14] Instituto de Investigaciones en Biodiversidad Argentina (PIDBA), Universidad Nacional de Tucumán (UNT). Tucumán, Argentina
[15] Instituto de Investigaciones Forestales y Agropecuarias Bariloche (IFAB) IFAB INTA-CONICET, Agencia de Extensión Rural de El Bolsón
[16] Institute of Geographical Sciences, Freie Universität Berlin, Germany

* Corresponding author
E-mail: maria.piquer-rodriguez@fu-berlin.de

Methodological Considerations for Quantifying Author's Contributions

Our Women's Contribution Index (WCI) aims to reflect the degree of contribution to a paper in terms of time investment by the authors based on their positions in a co-authorship list. The main underlying assumption is that the more time invested by an author, the closer is its name to the first position. We agree that this assumption should be the baseline for deciding positions in an author list in almost every article. However, there could be other factors influencing as well, inducing alterations in such baseline order. The most controversial may be the last author, who is sometimes intendedly assigned to hierarchize senior researchers. As a group, we embraced this discussion during several meetings arguing whether it was possible to adapt the WCI to reflect to the best extent possible the situations across our dataset as a whole. We concluded that, given that there is not enough information to certainly unravel the role of all authors in all papers, any deviation from the simple harmonic allocation *sensu* Hagen (2008) [1] incorporates more problematic assumptions.

We consider it enriching to share this discussion with the readers, presenting a summary of the main insights through this appendix. In the first section, we enlist possible situations behind the inclusion and allocation of authors in a paper (Table S1). This overview of situations is not intended to be exhaustive, as there can be as many particular cases as there are paper projects. The second section is dedicated to elaborating on the case of the last author. Finally, in the third section, we discuss potential alternative adaptations of the WCI to particular cases.

Table S1. Situations behind the inclusion and allocation of authors in a paper.

Situation	Description	Reference
Ghost authors	The non-inclusion of someone who contributed to the papers. Commonly laboratory techniques or a statistician.	[2]
More than one senior author	The inclusion of more than one senior author may be co-authors in a paper. This phenomenon can be more frequent in longer lists of authorship.	[2]
Gift Authorship	The inclusion of an individual as a coauthor who didn´t contribute to the manuscript *per se*. This practice is also referred to as honorary authorship as a gesture. The laboratory group leader or another senior academic is prone to be gifted authors.	[2, 3, 4, 5, 6]
Guest Authorship / Forged Authorship	The inclusion of an influential researcher who "loans" their name to a study to enhance its credibility, usually as the last author. For instance, principal investigators frequently require that their names be included or listed first on research conducted within their department or laboratory.	[2, 3, 5, 6, 7]

Authorship in alphabetic order	The allocation of authors by their surnames in alphabetical order. Commonly employed in business academic publications. This strategy is usually used in multi-co-authorship papers behind the disciplines.	[8, 9, 10]
Institutional policies	The inclusion and allocation of authors often follow institutional policies related to covering the fees for open-access publishing. Some institutions require that their members be designated as the corresponding author, the first author, or the last author to cover the journal fees.	

2. The Last Author Issue

In this section, we present the rationale behind our methodological decision to keep the WCI as proposed by Hagen [1], without making special considerations for the last author. We believe this decision minimizes the likelihood of error in quantifying the contribution of the last author. Anyhow, we have categorized the potential errors based on how we assess the last author's contribution: either assuming the last author contributed more than the preceding authors (Type A) or assuming they contributed the least (Type B).

Type A Error:
This error occurs if we underestimate the last author's contribution, considering it poor when, in fact, they might be:
A.i: A senior author who has contributed at least more than the preceding author on the list.

Type B Error:
This error occurs if we overestimate the last author's contribution, considering it significant when they might be:
B.i: A gifted author (Table S1).
B.ii: A guest author (Table S1).
B.iii: The one who contributed the least.
B.iv: Someone positioned last due to their surname's initial letter being later in the alphabet than the preceding authors (Table S1).
B.v: An author randomly positioned last.

Given these scenarios, the most error-avoidant decision is to consider the last authors as having the least contribution. By not assuming that the last author is a senior author, we risk the opposite error: undervaluing their actual contribution. However, in cases where the last author is indeed a senior author, this issue is less severe since senior authors are typically very busy individuals whose time is divided among many projects. It is important to remember that the WCI aims to capture time investment.

The following items explain our arguments regarding the inconvenience of assuming that the last author consistently guides the research teams and contributes more than the previous one in the list.

1) Temporal trends regarding the last position occupancy. Our data are dated from 1999 to 2021. Historically, there was consensus that the contribution was decreasing along the list of authors. More recently the practice of the latest authors taking center stage, being praised as seniors, was incorporated. Currently, both strategies coexist. Considering the last author as a senior is not parsimonious enough. Duffy et al. [11] found in a survey that most ecologists view the last author as the “senior” author on a paper (i.e., the person who guides the research group in which most of the work was carried out). However, there was substantial variation in views on authorship, especially corresponding authorship. In 2016, the corresponding author was usually the first author (range across the four journals: 77%–90% of papers); less commonly, it was the last author (range across the four journals: 9%–18% of papers). The last author may or not reflect an advisory role [11, 12, 13]. Nevertheless, a co-authorship position may also imply other or even arbitrary decisions [14].

2) More than one senior author may be co-authors in a paper. In this situation, it is not easy (even impossible with our data) to inquire about the positions of multiple senior authors.

3) It is difficult to establish at what number of authors the last author becomes a significant contributor. We wonder at what number of authors does the last one become a considerable leader?

4) The Gift Authorship and Guest Authorship Problem: Laboratory group leaders or other senior academics are prone to be gifted authors. Authorship gifting happens when an individual is acknowledged in a study but doesn't meet the criteria for authorship. This practice is also referred to as honorary authorship. Essentially, it's a gesture; the individual doesn't qualify as an author per se. Guest authorship occurs when influential individuals "loan" their name to a study to enhance its credibility. Nevertheless, these individuals were not directly involved in the research itself. One of the main drivers of guest authorship stems from the hierarchical organization of contemporary laboratories. For instance, principal investigators frequently require that their names be included or listed first on research conducted within their department or laboratory. They assert this demand based on their acquisition of research funds or their provision of top-level supervision. Both Gift Authorship and Guest Authorship are related to the Matthew effect. Both gift and guest authorship are commonly accepted by the actual authors partly because it's widely understood that an article gets benefits in terms of acceptance if they include the name of a renowned researcher. This situation adds extra noise to our attempts to understand roles in academia given that many laboratory heads and senior academics are male, they are overrepresented given the glass ceiling of the leaky pipeline.

In sum, we prefer to avoid an allocation of weights that involves a greater number of additional assumptions that could obscure the data trends

3. Discussing alternative adaptations of the WCI to particular cases

Our approach follows the Harmonic Allocation of Authorship Credit proposed by Nils Hagen in 2008 [1] for measuring the contribution of multiple authors in a paper. What we contribute to building the WCI is tallying only the contributions made by female authors; in other words, other authors might employ different methods to measure contributions in multi-authored papers and calculate the WCI. We invite future studies to propose enhancements in index construction. As possible correction can still use some version of the Harmonic Allocation of Authorship Credit [1].

If we assume that the last author is the team leader of a particular paper: Which would be the best way to value their weight? Equal to the first author? Equal to the second author? Both

possibilities may be ok. Another possibility could be to consider the weight of the last author as the average weight of all other authors, and then distribute the remaining weight among the remaining authors using harmonic decay.

Hagen [1] discusses the possibility of including additional byline information about the equality of some co-authors' contributions, or implicit information about the approximate equality of contributions by the first and last authors. Such variations are easily accommodated by a harmonic counting scheme with little or no alteration of the credit allocated to the remaining coauthors (see Hagen Figure 5). Although the Harmonic Allocation of Authorship Credit can deal with, for example, first and last authors equally merited (proposed by Hagen itself), we still think our study case prevents us from doing so. For further clarifications see arguments in Hagen [1].

We agree that the corresponding author information is valuable. Frequently used to indicate in which situations the last author is the team leader. Unfortunately, we did not register them in our data set. Nevertheless, due to Duffy [11] finding that 84% of papers published in 2016 had the first author as the corresponding author, we think that our results are somehow capturing the corresponding author's trends. We encourage future research to include considerations about corresponding author and credit taxonomy in new versions of the Women Contribution Index.

References

1. Hagen NT. Harmonic Allocation of Authorship Credit: Source-Level Correction of Bibliometric Bias Assures Accurate Publication and Citation Analysis. PLoS ONE. 2008;24;3(12):e4021. doi: 10.1371/journal.pone.0004021

2. Tarkang EE, Kweku M, Zotor FB. Publication Practices and Responsible Authorship: A Review Article. J Public Health Afr. 2017 27;8(1):723. doi: 10.4081/jphia.2017.723

3. McNutt MK, Bradford M, Drazen JM, Hanson B, Howard B, Jamieson KH, et al. Transparency in authors' contributions and responsibilities to promote integrity in scientific publication. Proc Natl Acad Sci. 2018;115(11):2557-2560. doi: 10.1073/pnas.1715374115

4. Al-Herz W, Haider H, Al-Bahhar M, Sadeq A. Honorary authorship in biomedical journals: how common is it and why does it exist? J Med Ethics. 2014;40(5):346-348. doi: 10.1136/medethics-2012-101311

5. Rennie D, Flanagin A. Authorship! Authorship! Guests, Ghosts, Grafters, and the Two-Sided Coin. JAMA. 1994;271(6):469–471. doi: 10.1001/JAMA.1994.03510300075043

6. Anstey A. Authorship issues: grizzles, guests and ghosts. Br J Dermatol. 2014;170(6):1209-1210 doi: 10.1111/bjd.13095

7. Merton RK. The Matthew Effect in Science. Science. 1968; 159,56-63. doi: 10.1126/science.159.3810.56

8. Fernandes JM, Cortez P. Alphabetic order of authors in scholarly publications: a bibliometric study for 27 scientific fields. Scientometrics. 2020;125:2773-2792. doi: 10.1007/s11192-020-03686-0

9. Ackerman M, Brânzei S. The authorship dilemma: alphabetical or contribution? Auton Agent Multi-Agent Syst. 2017;31:1077-1093.1. doi: 10.1007/s10458-016-9351-7

10. Joanis ST, Patil VH. Alphabetical ordering of author surnames in academic publishing: A detriment to teamwork. PLoS ONE. 2021;16(5):e0251176. doi: 10.1371/journal.pone.0251176

11. Duffy MA. Last and corresponding authorship practices in ecology. Ecology and Evolution.

12. Wager E. Recognition, reward and responsibility: Why the authorship of scientific papers matters. Maturitas. 2009 Feb;62(2):109–12.

13. Ross MB, Glennon B, Murciano-Goroff R, Berkes E, Weinberg BA, Lane J. Women are credited less in science than men. Nature. 2022 Jun 22;608(7921):135–45.

14. Larivière V, Pontille D, Sugimoto CR. Investigating the division of scientific labor using the Contributor Roles Taxonomy (CRediT). Quantitative science studies. 2021 Jan 1;2(1):111–28.
