# Supplementary material for: Over twenty years of publications in Ecology: Over-contribution of women reveals a new dimension of gender bias
Source: PLoS One. 2024 Sep 19;19(9):e0307813. doi: 10.1371/journal.pone.0307813 (PMC11412523; doi:10.1371/journal.pone.0307813)
Supplement: S3 File — (PDF) [file pone.0307813.s003.pdf]

Alternative Language Abstract (Spanish)
Supporting Information for:

Over twenty years of publications in Ecology: Over-contribution of Women reveals a new dimension of gender bias

Gabriela Fontanarrosa[1¶], Lucía Zarbá[2¶], Valeria Aschero[3], Daniel Andrés Dos Santos[1, 4], M. Gabriela Nuñez Montellano[5], Maia C. Plaza Behr[5], Natalia Schroeder[6,7], Silvia Beatriz Lomáscolo[5], María Elisa Fanjul[4,8], A. Carolina Monmany Garzia[5], Marisa, Alvarez[9,10], Agustina Novillo[1], María José Lorenzo Pisarello[11], Romina Elisa D'Almeida[12], Mariana Valoy[8], Andrés Felipe Ramírez-Mejía[5], Daniela Rodríguez[6,7], Celina Reynaga[1], María Leonor Sandoval Salinas[13,14], Verónica Chillo[15] & María Piquer-Rodríguez[16] *

[1] Instituto de Biodiversidad Neotropical (IBN), Consejo Nacional de Investigaciones Científicas y Técnicas (CONICET), Facultad de Ciencias Naturales e Instituto Miguel Lillo, Universidad Nacional de Tucumán (UNT), Yerba Buena, Tucumán, Argentina
[2] Instituto de Investigaciones Territoriales y Tecnológicas para la Producción del Hábitat UNT-CONICET, Tucumán, Argentina.
[3] Instituto Argentino de Nivología, Glaciología y Ciencias Ambientales (IANIGLA), CONICET, Universidad Nacional de Cuyo (UNCuyo), Argentina
[4] Instituto Vertebrados, Zoología, Fundación Miguel Lillo. Facultad de Ciencias Naturales e Instituto Miguel Lillo. Universidad Nacional de Tucumán, Argentina.
[5] Instituto de Ecología Regional (IER), Universidad Nacional de Tucumán (UNT)- Consejo Nacional de Investigaciones Científicas y Técnicas (CONICET), Tucumán, Argentina
[6] Instituto Argentino de Investigaciones de las Zonas Áridas (IADIZA), CCT-CONICET
[7] Facultad de Ciencias Agrarias, Universidad Nacional de Cuyo, Mendoza, Argentina
[8] Fundación Miguel Lillo, Tucumán, Argentina
[9] Universidad Nacional de Tucumán, Argentina (UNT)
[10] Universidad Nacional de Santiago del Estero, Argentina (UNSE)
[11] Centro de Referencia para Lactobacilos CCT NoA Sur. Consejo Nacional de Investigaciones Científicas y Técnicas (CONICET)h

[12] Instituto Superior de Investigaciones Biológicas (INSIBIO). CCT NoA Sur. Consejo Nacional de Investigaciones Científicas y Técnicas (CONICET)
[13] Instituto de Investigación en Luz, Ambiente y Visión (ILAV), CONICET-UNT
[14] Instituto de Investigaciones en Biodiversidad Argentina (PIDBA), Universidad Nacional de Tucumán (UNT). Tucumán, Argentina
[15] Instituto de Investigaciones Forestales y Agropecuarias Bariloche (IFAB) IFAB INTA-CONICET, Agencia de Extensión Rural de El Bolsón
[16] Institute of Geographical Sciences, Freie Universität Berlin, Germany

* Corresponding author
E-mail: maria.piquer-rodriguez@fu-berlin.de

Más de veinte años de publicaciones en Ecology: la sobrecontribución de las mujeres revela una nueva dimensión del sesgo de género

Las características biográficas como el estatus social y económico, la etnicidad, la sexualidad, los roles de cuidado y el género perjudican injustamente a los individuos dentro del ámbito académico. Los patrones de autoría deben reflejar la dimensión social detrás del proceso de publicación y la dinámica de coautoría. Para detectar posibles sesgos de género en la autoría de artículos y examinar la magnitud de la contribución de las mujeres en términos del volumen sustancial de producción científica en Ecología, investigamos artículos de la revista de mayor rango, Ecology, desde 1999 hasta 2021. Desarrollamos un Índice de Contribución de las Mujeres (WCI, por sus siglas en inglés) para medir las contribuciones individuales basadas en el género. Considerando el género, la posición en la lista de autores y el número total de autores, el WCI calcula la suma de la contribución de cada mujer por artículo. Comparamos el WCI con las contribuciones esperadas de las mujeres en un escenario sin sesgo de género. En general, las mujeres representan el 30% de los autores en Ecology, pero su contribución a los artículos es mayor de lo esperado por casualidad (es decir, una sobrecontribución). Además, al comparar el WCI con un Índice de Contribución de los Hombres equivalente, encontramos que las mujeres consistentemente tienen mayores contribuciones en comparación con los hombres. También observamos una tendencia temporal de aumento en la autoría de mujeres y en los artículos de género mixto. Esto sugiere algunos avances en la lucha contra el sesgo de género en el campo de la ecología. Sin embargo, enfatizamos la necesidad de una mejor comprensión del patrón de sobrecontribución, que puede derivarse parcialmente del fenómeno de la sobrecompensación. En este contexto, las mujeres podrían necesitar superar a los hombres para ser percibidas y evaluadas como iguales. El WCI proporciona una herramienta valiosa para cuantificar las contribuciones individuales y entender los sesgos de género en la publicación académica. Además, el índice podría personalizarse para adaptarse a la pregunta específica de interés. Sirve para descubrir un tipo de sesgo previamente no cuantificado (la sobrecontribución) que,

argumentamos, es la respuesta a la estructura inequitativa del sistema científico, lo que lleva a diferencias en los roles de los individuos dentro de un equipo de publicación científica.
